# The effect of prosthetic feedback on the strategies and synergies used by vestibular loss subjects to control stance

**DOI:** 10.1186/1743-0003-10-115

**Published:** 2013-12-19

**Authors:** Flurin Honegger, Imke MA Hillebrandt, Nadja GA van den Elzen, Kok-Sing Tang, John HJ Allum

**Affiliations:** 1 Department of ORL, University Hospital, Petersgraben 4, CH - 4031 Basel, Switzerland; 2 Department of Neurology, Radboud University Medical Centre, Nijmegen, The Netherlands; 3 Biomaterials Science Center (BMC), University of Basel, Basel, Switzerland

**Keywords:** Prostheses and Implants, Feedback/physiology, Vestibular diseases, Postural Balance/physiology, Electromyography

## Abstract

**Background:**

This study investigated changes in stance movement strategies and muscle synergies when bilateral peripheral vestibular loss (BVL) subjects are provided feedback of pelvis sway angle.

**Methods:**

Six BVL (all male) and 7 age-matched male healthy control (HC) subjects performed 3 stance tasks: standing feet hip width apart, eyes closed, on a firm and foam surface, and eyes open on foam. Pelvis and upper trunk movements were recorded in the roll and pitch planes. Surface EMG was recorded from pairs of antagonistic muscles at the lower leg, trunk and upper arm. Subjects were first assessed without feedback. Then, they received training with vibrotactile, auditory, and fall-warning visual feedback during stance tasks before being reassessed with feedback.

**Results:**

Feedback reduced pelvis sway angle displacements to values of HCs for all tasks. Movement strategies were reduced in amplitude but not otherwise changed by feedback. These strategies were not different from those of HCs before or after use of feedback. Low frequency motion was in-phase and high frequency motion anti-phasic. Feedback reduced amplitudes of EMG, activity ratios (synergies) of antagonistic muscle pairs and slightly reduced baseline muscle activity.

**Conclusions:**

This is the first study demonstrating how vestibular loss subjects achieve a reduction of sway during stance with prosthetic feedback. Unchanged movement strategies with reduced amplitudes are achieved with improved antagonistic muscle synergies. This study suggests that both body movement and muscle measures could be explored when choosing feedback variables, feedback location, and patient groups for prosthetic devices which reduce sway of those with a tendency to fall.

## Background

Loss of vestibular function is well-known as a factor underlying an increased tendency to fall in older persons [1]. Furthermore, a number of persons with less than 60 years of age, having either a unilateral or bilateral peripheral vestibular loss (UVL or BVL) have difficulty maintaining their balance particularly for complex gait tasks such as climbing stairs, or for stance tasks for which visual and proprioceptive inputs are reduced, such as standing on a carpet in the dark [2]. For these reasons, a number of investigators have developed balance prostheses to provide persons with balance problems a replacement for vestibular sensory information on body sway. Such prosthetic systems generally rely on vibrotactile or auditory feedback or both modes, appropriately coded with body sway information [3-9]. There are variations concerning where the sway measures are taken, and how the sway signals are processed, and at which body location the feedback is provided. However, the general conclusion is that such feedback helps UVL and BVL patients improve their balance during stance and gait [3,7,9].

There are different opinions on whether position or velocity feedback should be used for balance prostheses. Early results suggest that angle position feedback was more effective than velocity feedback of pelvis sway [6] and, when employed, led to reduction of angle rather than velocity of sway [5]. Others have used a combination of angle and velocity feedback with success [7-11]. Another crucial question is whether, for effective sway reduction, patients need to use a particular type of movement strategy. One way to begin answering this question is to examine a patient group such as those with vestibular loss that have similar but exaggerated movement strategies to those of healthy controls (HCs)in contrast to those with proprioceptive loss [12]. For HCs, feedback of pelvis sway angle is known to be effective in improving balance control [4,7].For these reasons, it has been suggested that subjects with vestibular loss might be more responsive to feedback modes that function well for healthy controls [13].

Thus the first question this study sought to answer was whether improvements in balance control achieved by vestibular loss subjects using artificial sway position feedback were brought about using the same movement strategies as when no feedback was present. This question is by no means as simple as considering body sway during stance as similar to that of an inverted pendulum, because the upper and lower parts of the body move with two modes simultaneously during stance [12,14]. One mode, a low frequency (<0.7 Hz) mode, is like a “floppy” inverted pendulum with mostly in-phase motion of the pelvis and trunk. The other, high frequency (> 3 Hz) mode, is an anti-phase motion of the 2 segments. Between 0.7 and 3 Hz, motion transitions between these 2 modes. These movement strategies remain similar when the postural task conditions change, for example if the eyes are closed or open or if the support surface is firm or of foam [12]. The characteristics of these movement strategies suggest that the muscle synergies possibly underlying reductions in sway amplitudes for these two modes must at least be driven by muscles acting at the ankle joints and at the trunk. If as shown by Goodworth et al. [9], improvements are only present in the low-frequency inverted-pendulum mode of motion, then changed control of muscle activity at the ankle joint would be sufficient for better balance. The typical changes in muscle synergies observed with vestibular loss in response to rotational perturbations of the support surface cause changes in both modes of motion described above for control of quiet stance [15]. These changes consist of reduced ankle muscle activity but increased trunk muscle activity with respect to responses of healthy controls [16,17]. Thus the second question, and most important question we have attempted to answer with this study is how muscle synergies are changed when feedback of pelvis sway angle is provided to BVL subjects.

## Methods

### Participants

Thirteen adult subjects (6 bilateral peripheral vestibular loss (BVL) subjects and 7 age-matched healthy control subjects) were included in this study. The BVL subjects were out-patients at the University Hospital of Basel, Switzerland. The BVL subjects ranged from 45 to 52 years of age (Mean 48.8; SD 2.2) and the controls ranged from 41 to 53.2 years (Mean age 49.2 SD 4.4). Inclusion criteria for the BVL subjects was the absence of vestibular ocular reflex responses to caloric irrigation with water temperatures of 44 and 30 deg C for both ears, rotating chair (yaw and pitch) responses less than the lower 5% bound of normal subjects. All BVL subjects had normal auditory evoked potentials and normal pure tone audiograms (PTA) except one subject whose PTA bone conduction thresholds were at 70 dB hearing level. All BVL subjects had normal magnetic resonance imaging scans of the brain. The BVL subjects had no other neurological or musculoskeletal impairments that could interfere with balance control. Exclusion criteria for the healthy subjects included self-reported sensory, neurological or musculoskeletal impairments that could interfere with balance and inability to stand on one leg, eyes closed, for 20 seconds without falling. All subjects provided informed written consent to participate as required by the ethical committee of the University Hospital of Basel who approved the study.

### Testing procedures and data recording

**Figure 1:**
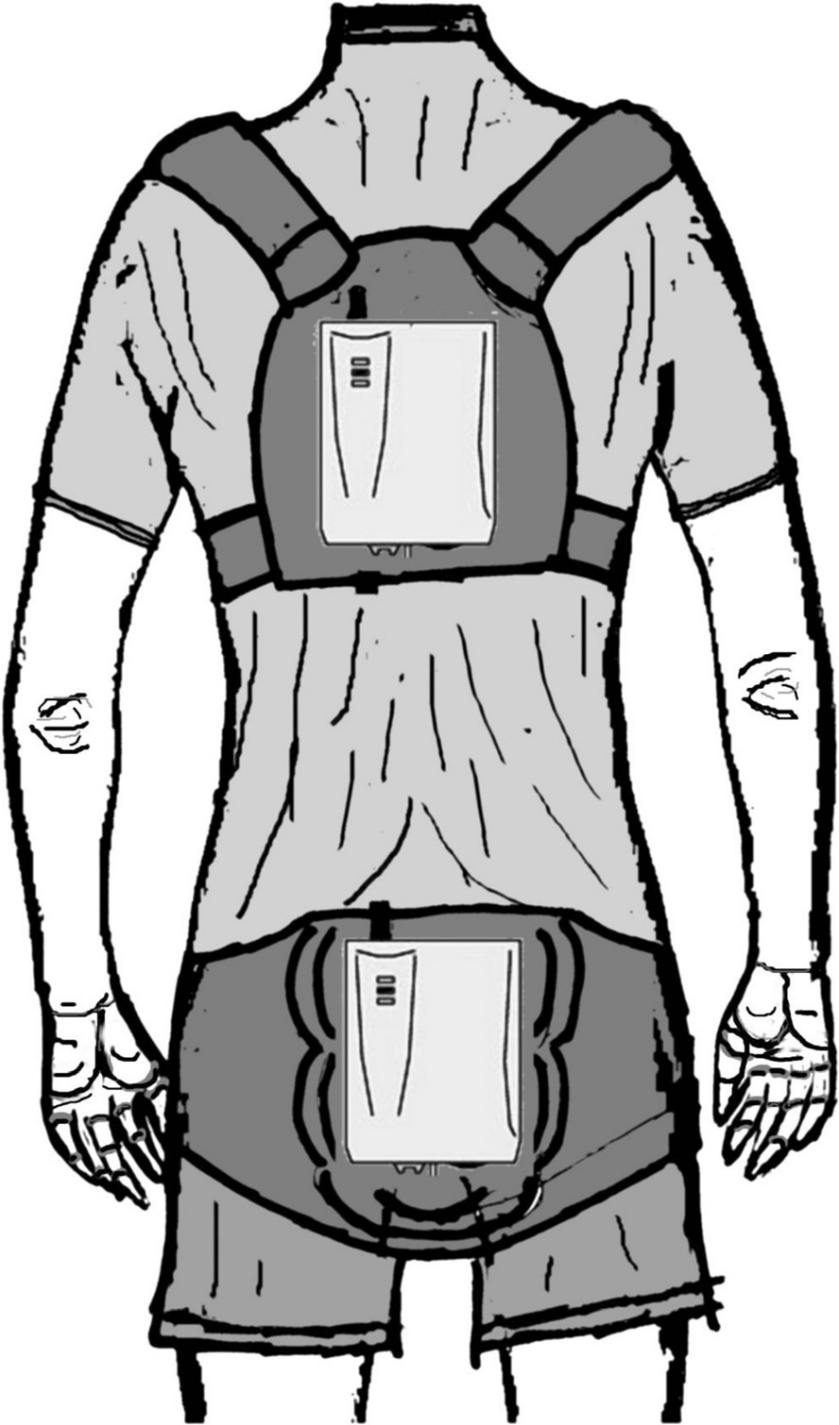
Schema showing the placement of the trunk and pelvis gyroscopes on the subject’s back.

**Figure 2:**
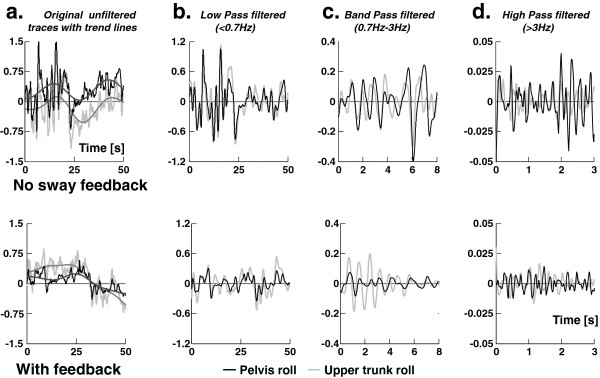
**Improvement in roll sway for a BVL subject when provided feedback of pelvis sway angle.** The upper four panels show the sway angles in degrees of the pelvis and upper trunk while standing feet shoulder width apart, eyes closed on foam without feedback, the lower panels with feedback. **a**. 50 seconds of the original unfiltered traces of upper trunk and pelvis sway with general trend lines. **b**. The same traces as in a. after removing the general trend and low pass filtering. **c**. 20 seconds of the same traces as in a. after removing the general trend and band pass filtering. **d**. 5 seconds of the same traces as in a. after removing the general trend and high pass filtering.

**Figure 3:**
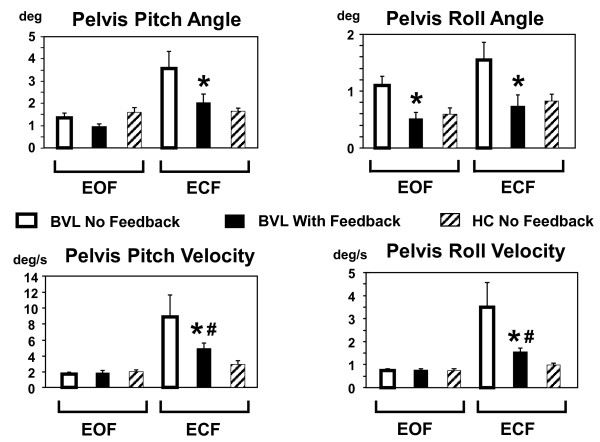
**Group angle and angular velocity means of 90% pelvis sway ranges with and without feedback.** The column height represents the mean group pelvis angle or angular velocity for each task. Values are shown for stance tasks on foam with eyes open (EOF) or closed (ECF). The vertical lines above each column indicate the standard error of the means. BVL stands for bilateral vestibular loss group, HC for the healthy control group. BVL means with feedback marked with * have a significant decrease compared to means without feedback. If the means of BVL subjects with feedback remained significantly greater than healthy controls the BVL values are marked with #.

Two gyroscope based systems, SwayStar (Balance International Innovations GmbH, Switzerland), measuring angular velocities in the roll and pitch planes, were used. The gyroscopes typically had a bias drift of 1–2 deg/h, manufacturer’s specification 6 deg/h. One SwayStar system was mounted on a belt strapped around the hips in order to measure pelvis movements. The other system was mounted on a plate placed between the scapulae and held in place with a tightly fitting shoulder harness (see Figure 1). The angular velocities were sampled at 100 Hz with 16 bit accuracy over a range of 327 deg/s and then transferred wirelessly to a PC which computed angle changes via trapezoid integration [18]. Because sensor drift was low, no correction was made for drift other than that described in data processing below. Muscle activity was measured with pairs of surface, silver-silver chloride EMG electrodes. These electrodes were placed 3 cm apart, along several muscles on the left side of the body: tibialis anterior, soleus, external obliques, paraspinalis at L4-L5, and the medial deltoid. We recorded only left-sided responses as we assumed instability would be predominately in the symmetrical pitch direction (see Figures 2 and 3). EMG signals were recorded using a preamplifier with a gain of 1000 and a band-pass of 0.7 Hz to 2.5 kHz. The signals were analog band-pass filtered between 60 and 600 Hz, full-wave rectified and low-pass filtered at 100 Hz with a 3rd order Paynter filter prior to sampling at 1 kHz [19].

A BalanceFreedom system (Balance International Innovations) provided feedback of pelvis sway angle to the participants in the form of vibrotactile, auditory and visual signals using as the feedback input the computed pelvis angles obtained from the pelvis gyroscopes angular velocity outputs. Actuators for the feedback were mounted on a head band and were active once pelvis sway angle thresholds for activating respectively the vibrotactile, auditory, and visual actuators were exceeded. We chose to use three types of feedback to indicate increasing sway rather than 3 rows of vibrators in a column as Wall and coworkers have done [10] because of the evidence that both vibrotactile and auditory feedback provide improvements in sway [4,6,8,11]. The visual feedback with the highest threshold was conceived as a flashing warning of a possible fall. Furthermore the actuator system was placed on the head rather than around trunk muscles at the waist to avoid possible interactions with muscle proprioception. Feedback thresholds were based on individual values of the 90% ranges of pelvis sway in the pitch and roll directions computed for the 70 sec duration (or less) of each task of the first assessment’s sequence of tasks described below. The thresholds in each direction were set at ±40% of the 90% range for vibrotactile signals (that is, a range equal to 80% of the 90% measurement), ±80% for the acoustic signals and ±150% for the visual threshold. This meant that, for example, the vibrotactile thresholds were equal on average to 40% of the roll and pitch mean angle values shown in Figure 3 (no feedback or first assessment columns). Thus the values set for the BVL subjects were significantly larger for the eyes closed foam task (see below for task descriptions) than would have been set for the HCs. Once activated, each feedback signal remained active as long as its threshold was exceeded with the acoustic feedback increasing linearly in amplitude from 50 dB until it reached 70 dB hearing level at the visual threshold. The vibrotactile activation signal was sent to 1 of 8 vibrators in the headband set at 45˚ intervals around the headband. A vibrator switched on when the sway threshold was reached in the direction of the sway and this direction had the largest sway amplitude. The frequency of vibration was 150 Hz. This is in the middle of the frequency range of maximum sensitivity of skin receptors to vibration [20]. The acoustic feedback consisted of two bone-conducting acoustic actuators placed above the ears at the level of the mastoids. The left actuator was activated at 870 Hz when the acoustic threshold was reached for sway to the left, the right actuator at 500 Hz when swaying to the right, and both conductors with a frequency of 1370 Hz and 250 Hz when swaying backwards and forwards, respectively. The visual feedback served as a flashing warning signal regardless of sway direction [5].

For each of the two assessments first without and then with feedback, two stance tasks were performed on a foam surface, eyes open and eyes closed and one task eyes closed on a normal surface. The foam surface had a height of 10 cm, length 100 cm, width 44 cm and a density of 25 kg/m^3^. We used a foam surface because this reduces the effectiveness of lower leg proprioceptive inputs to control balance and makes it very difficult for vestibular loss subjects to control their balance, especially with eyes closed [2,12]. Pelvis and trunk sway and EMG signals were recorded for 70 seconds during each of the stance tasks. If a loss of balance occurred during a task, the task was repeated a maximum of two times and the trial with the largest duration was used. The tasks were performed without shoes, feet hip width apart, and with the arms hanging alongside the body. After the first assessment, subjects rested for 20 minutes. Then, 30 minutes of training was provided with biofeedback. The training tasks were the same as those of the first assessment but also included tandem stance on a firm surface, eyes open and closed so that difficult training tasks were also provided. After another short pause of 5 minutes, subjects were reassessed on the same 3 stance tasks of the first assessment with the feedback active. During all tasks, two spotters stood close behind the subjects in order to prevent a potential fall.

### Data processing and statistical analysis

Angle data was obtained by integrating the recorded angular velocity data. The low frequency trend in the angle data was determined by applying the dynamically parameterized “denoise” function of the “Rice Wavelet Toolbox” [21] to the angle data and subtracting this denoised data from the original angle data. The resulting data were subsequently filtered and thereby separated into three frequency bands (see Figure 2): low pass (<0.7 Hz), high pass (>3.0 Hz) and band pass (0.7-3.0 Hz). The filtering is based on previous observations of power spectral densities (PSDs) of pelvis sway [12] – two resonances are observed, one below 0.7 Hz, the other above 3 Hz. This filtering was implemented using simple 3rd order Butterworth filters running forwards and backwards over the data.

**Figure 4:**
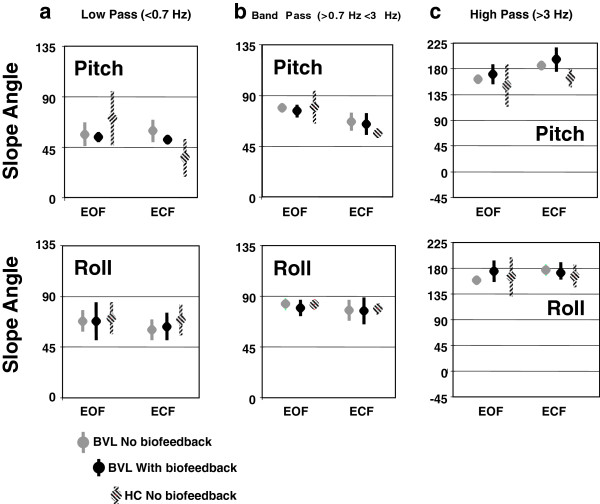
**Mean regression slopes of trunk angles with respect to pelvis angles.** Mean regression slopes are depicted for the two foam tasks, eyes open and closed, without and with biofeedback. The upper panels show the slope angles in pitch, the lower panels in roll. **a**. slope values for low-pass filtered angle values, **b**. band-pass filtered angles and **c**. for high-pass filtered values. The bullet symbol marks the mean value of the slope; the vertical line depicts the 95% confidence intervals of the mean.

To measure pelvis-trunk coordination, total least-squares regression lines [22] were calculated in each frequency band with respect to the x-y plots of trunk versus pelvis angle samples for the roll and pitch data (see Figures 2 and 4). The slope angles (gradients) of the regression lines were further processed and visualized by applying circular statistics [23]. For statistical calculations the “Circular Statistic Toolbox” update 2010b published by P. Behrens [24] was used. For the graphical representation (Figure 4) negative slope angles where mapped to the corresponding positive 2^nd^ quadrant. Slope angles represent axial data and are bimodal because for example -70° and 110° represent the same data point (tan (*a*) = tan (*a* + 180)). Differences in slope regression angles between groups and conditions were examined with the Harrison-Kanji test which is the circular analogue of a two-factor ANOVA. Posthoc differences in group means for each condition were examined with a Watson-Williams multi-sample test for equal means after testing for value directedness and concentration (Rao and Rayleigh tests).

With the help of the MATLABs Signal Processing Toolbox Version 6.13 (R2010A) we calculated power spectral densities (PSDs) and PSD ratios for the EMG data (after 100 Hz low-pass filtering) data. Fast Fourier transformation was performed on a window size of 2048 samples (20.5 s) with an overlap of 1024 samples. Shifts in EMG baseline activity due to feedback were examined by considering the first quartile - 25% - values (Q1) of EMG amplitude samples. Changes in EMG amplitude modulation (mV) were investigated using the 90% ranges of EMG samples. Data of near falls at the end of the recordings were associated with large changes in EMG activity and possible arm movements were excluded from the analysis.

Because neither Q1 values nor the PSD of EMG data were normally distributed, differences in PSDs and PSD ratios (the ratio of paraspinals to obliques and tibialis to soleus as a measure of coactivation at the trunk and ankle joint muscles) between the two groups, BVL (with and without feedback) and controls across stance conditions (stance tasks) were analysed depending whether the situation required a paired or independent samples test using either the non-parametric Mann–Whitney or the Wilcoxon Signed Ranks test. To examine PSD amplitudes and ratios, the data were averaged over 19 frequency bins that combined values of 3 adjacent frequency samples. These bins are equally spaced on a logarithmic scale across the range of 0.00 – 40.04 Hz. The bins were: 1.95 (1.46-2.44 Hz), 2.44 (1.95-2.93),.2.93 (2.44-3.42), 3.91 (3.42-4.39), 4.39 (3.91-4.88), 4.88 (4.39-5.37), 6.84 (6.35-7.32), 8.30 (7.81-8.79), 9.77 (9.28-10.25), 11.23 (10.74-11.72), 13.18 (12.70-13.67), 15.63 (15.14-16.11), 18.07 (17.58-18.55), 21.00 (20.51-21.48), 24.90 (24.41-25.39), 29.30 (28.81-29.79), 34.18 (33.69-34.67), 40.04 (39.55-40.53). For all statistics p ≤ 0.05 was defined as significant, with corrections for multiple comparisons as necessary.

## Results

The pelvis sway feedback helped BVL subjects reduce sway angles at the pelvis and the upper trunk. Figure 2 provides an example of the improvement for a typical BVL subject who had considerable difficulty to stand eyes closed on the foam surface (ECF) particularly in the roll direction. The vibrotactile roll feedback was on for 33% of the trial. Pitch vibrotactile pitch feedback was active 6% and auditory feedback was not active in this trial. Figure 3 shows the mean BVL group 90% ranges of sway angle and angular velocity at the pelvis for the eyes closed and open on foam compared to mean values of the healthy controls (HC). There was a BVL group improvement with feedback which was significant for all ECF angle and angular velocity measures, There was also an improvement in roll angle eyes open on foam (EOF) and eyes closed on a normal surface (ECN) Below we focus our analysis mainly on the ECF condition because this task is the most difficult for the BVL subjects and showed the greatest improvement.

As shown in Figure 3, the sway angle amplitudes for the pelvis of all BVL subjects in roll and pitch were significantly reduced with feedback under ECF conditions to levels that were not different from those of HCs. Sway velocities standing ECF were reduced with feedback, but levels were still greater than those of HCs (Figure 3). There was a change in roll angles when BVL subjects stood with feedback EOF or ECN to the levels of HC. Other measures for these later conditions were not significantly different from those of healthy controls without feedback. When angular displacements of the trunk and pelvis for the ECF condition were split into different frequency bands, the most consistent improvements across frequencies were noted for the roll direction (significant reduction for all bands except a trend for trunk roll). Nonetheless, a trend for pitch improvements occurred in several frequency bands. However, these trends were only significant for two frequency bands (<0.7 Hz trunk and >3Hz pelvis). Thus for both roll and pitch improvement was not only limited to low frequency bands (<0.7 Hz).

### Movement strategies

**Figure 5:**
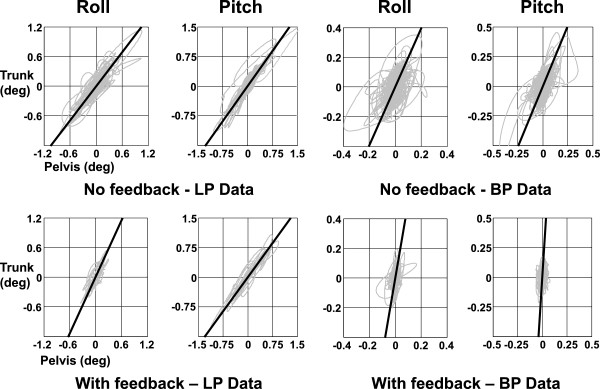
**Regression slopes of trunk versus pelvis movements of a BVL subject standing eyes closed on foam with and without feedback.** Regression slopes after low-pass filtering (LP) and band-pass filtering (BP) of trunk and pelvis sway angle traces are shown. The upper panel shows the slopes without biofeedback, the lower ones with feedback. On the left are the low pass regressions, on the right the band-pass regressions.

Phases representing movement strategies present between the pelvis and upper trunk were examined using correlation plots of pelvis and trunk angle divided into low (<0.7 Hz), middle (0.7 to 3 Hz), and high (>3 Hz) frequency bandwidths. Perfectly in-phase movement-strategies between the trunk and pelvis would imply that the upper body moved as an inverted pendulum and correlation plots would have slope lines of 45°. The example low pass (LP) plots of Figure 5 indicate near in-phase low frequency movements, with pitch more in phase than roll. This trend is confirmed by the group values shown in Figure 4a. The mid-frequency (0.7 to 3 Hz) movements were still in-phase but are restricted to mostly trunk movements on a fixed pelvis (see Figures 4b and 5 right). High frequency (>3 Hz) movements were characterized by anti-phase motion (regression lines values greater than 90°). When BVL group values of the phase relationships were examined in each frequency band without and with feedback, no changes in these phase characteristics were observed when feedback was provided. Furthermore, the phase relationships did not differ from those of HCs.

### Muscle synergies

**Figure 6:**
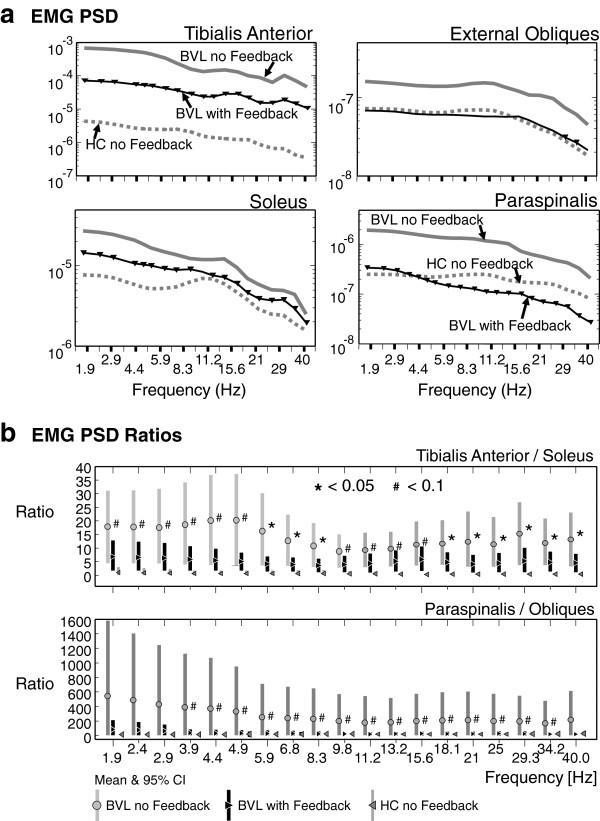
**EMG activity with and without feedback. a**: Mean group PSDs of BVL EMG activity with and without feedback compared to mean activity of controls without feedback for the task of standing eyes closed on foam. The upper grey line is mean BVL activity without feedback, the lower black line is the mean activity with feedback. Triangles indicate those frequency bins where the PSD values were significantly lower with feedback (p < 0.05). The grey dotted lines indicate the mean of healthy control values (without feedback). The mean values of BVL subjects with feedback were not significantly different from controls, except for tibialis anterior. **b**: Mean group activation ratios of pairs of ankle (tibialis/soleus) and trunk (paraspinals/obliques) muscles compared for BVL subjects with and without feedback and to controls. The ratios at each frequency are of PSD values shown at the same frequency in **a**. For example, the tibilalis anterior/soleus ratio for BVLs at 1.9 Hz is the ratio of the PSD values of the thick lines in A for tibialis and soleus. The vertical bars mark the 95% confidence intervals. Asterisks (*) mark significant (p < 0.05) differences of BVL subjects with and without feedback, while gate symbols (#) mark trends (p < 0.1).

**Figure 7:**
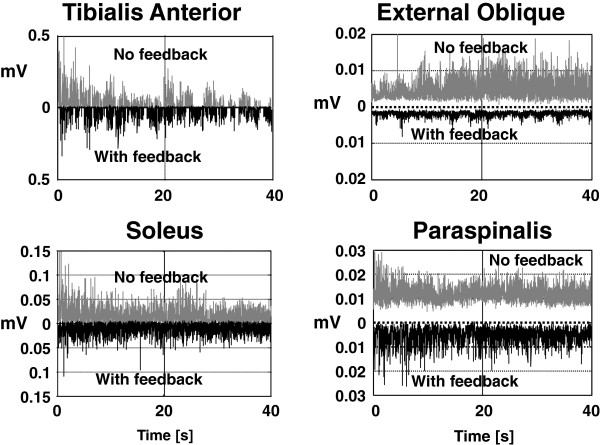
**EMG activity of a BVL subject with and without feedback for the task standing eyes closed on foam.** The ankle and trunk muscle activity without feedback is shown in the upper traces of each panel, the activity from the same muscles with feedback is shown inverted in each panel.

The data of BVL subjects illustrated in Figures 2, 3, 4 and 5 is consistent with a decrease in amplitude modulation when sway feedback is provided rather than any changes in movement strategies. The question arises how these changes in amplitude modulation are brought about. The patterns of underlying muscle activity shown in Figure 6 suggest three mechanisms. One mechanism is a change in the depth of modulation of muscle activity as depicted by the example in Figure 7 (left). The change in amplitude modulation across frequencies was examined with PSDs of the filtered EMG activity compared with and without feedback (see Figure 6a). A significant reduction of activity was observed for arm, ankle and trunk muscles across almost all frequency bands except for external obliques for which only a trend (p < 0.1) was observed. Figure 6a shows the mean group values for the ankle and trunk muscles. Although there were slight differences between BVL PSD values with feedback and those of healthy controls without feedback (see, for example, paraspinals in Figure 7), those differences were not significant, except for the tibialis anterior and deltoid muscles. These exceptions demonstrated lower activity levels in controls (Figure 6a). A second manner in which pelvis and trunk movements could be reduced is by lowering the general level of muscle activity. For this purpose, we used the median level lower 25% of the EMG histograms as a measure of the baseline EMG activity. With feedback, these levels were significantly reduced in the paraspinal and deltoid muscles for ECF conditions. The third method for muscle synergies to change would be in coactivation ratios. To examine these ratios we, used the PSD ratios of ankle and trunk muscle activity, as shown in Figure 6b. There was a trend for the ratio of paraspinals to obliques and tibialis to soleus to be reduced with feedback for ECF conditions. This trend was significant across several frequency bins for the ratio of tibialis anterior to soleus (for details see Figure 6b). Interestingly, the variances of BVL ratios were markedly smaller with feedback, however, still larger than those of HCs.

## Discussion

The results of this study indicate that for sway frequencies up to 10 Hz (the high frequency limit of our movement analysis) combined vibrotactile and auditory feedback leads to improved control of balance for BVL subjects. The improvement was present in both the roll and pitch directions. Our previous research on the direction sensitivity of feedback on balance control during stance has indicated that improvements are generally larger in the pitch direction for healthy subjects but are dependent on the amount of sway [5]. Therefore we assume that bilateral vestibular loss subjects benefit most by reducing their pitch sway about the pelvis as movements are larger in this direction. We have previously indicated that vestibular loss subjects tend to overreact at the trunk in the pitch and roll directions [15], and provide insufficient pitch control about the ankle joint [16]. Furthermore, differences in sensory processing times in these two directions may underlie the poor balance control in the roll direction [25]. Despite changes in movement amplitudes with feedback, there was no change in balance-correcting movement strategies employed when feedback of pelvis sway was provided. Moreover, the same movement strategies were used with and without feedback as those used by normal subjects.

The question arises whether the BVL subjects would have changed their movement strategies with more training. We have not looked at whether long-term balance training with the feedback alters movement strategies. However, it is unlikely that strategies would have changed over time as the strategies of BVL subjects with the larger feedback thresholds than would have been set for HC were similar to those of controls without feedback. We assume that long-term training would have reduced the feedback thresholds to values of HCs as BVL values would be individually reset to lower values following reassessment with no feedback. Lower no feedback values would be achieved via a carry-over effect of the feedback to the no feedback condition [3].

The amplitudes of both the low frequency (0.7 Hz) in phase and the high frequency (>3 Hz) anti-phase movement strategies were reduced with pelvis sway feedback by employing three types of muscle action: reduced depth of activity modulation, reduced background activity, and reduced muscle activation ratios. Presumably reduced background activity reduces intrinsic muscle stiffness and therefore induces less extensive sway for the same muscle modulation. However, this action was reinforced by smaller muscle modulation and lower antagonistic activation ratios. To our knowledge, this is the first study to show a direct neural correlation with the improvements in sway using prosthetic feedback. The reduction in EMG activity correlated with sway improvement raises the interesting question whether EMG feedback would provide a better feedback parameter than pelvis angular position as used here. EMG feedback is widely used to control upper limb prostheses. However, force control is equally as effective in controlling prosthetic limb position [26]. Here we used pelvis position as the pelvis CoM is close to that of the whole body CoM and we assumed that this latter variable is controlled by the CNS. It may well be worthwhile considering in future studies a dual feedback mode (CoM position and EMG) for prosthetic feedback, particularly for gait tasks where excessive EMG activity may help predict gait instabilities in the next step.

The reduction in both movement amplitudes and EMG activity with feedback raises the interesting question: Is a reduction in sway brought about by an increase or a decrease in muscle activity? There are circumstances, anxiety brought about by height, where increased activity leads to less sway [27]. However there are circumstances where the opposite is true, for example, when large destabilizing 1^st^ trial responses habituate to lower muscle and body displacements [28]. Probably a modelling technique would be required to show that the reduced muscle activity with feedback causes reduced movements. In the absence of such modelling our assumption that the two are associated is the most parsimonious explanation of our results.

It was interesting to note that the deltoid activity was also reduced with feedback. Whether this reduction is a direct result of reduced use of the arms to help balance or a reduced startle effect when balance is being lost cannot be inferred from our results. The use of the arms to control body sway when perturbed has, however, a limited effect on the CoM movement [29]. The movement of the arms due to enhanced deltoid activity is both part of acoustic startle responses and first trial effects to support surface perturbations [28]. Thus we would assume that BVL subjects became more confident with feedback training and therefore less startled when they realised they might fall.

The reductions in sway we observed were mostly in sway angles, but as sway angle became larger under the more difficult sensory conditions with the task of standing eyes closed on foam, velocity reductions occurred as well. These rapid reactions to the feedback may even have been reflex-like responses as it is hard to envisage the improvements we noted for movements with content greater than 3Hz (see Figure 2d) and reduced EMG modulation up to 40 Hz (Figure 6b) being solely due to voluntary reactions. This raises the question about the best way to code sway information in a balance prosthesis in order to produce the most effective feedback. Goodworth et al. [9] indicated that vibrotactile feedback provides only low frequency (<0.6 Hz) information on sway. It has been argued that this result occurred because feedback parameters were not fitted individually, rather a group “one-fits-all” approach was used [30]. Our thresholds were determined individually. It is also possible that mainly position information is extracted by subjects from our vibrotactile feedback signals and velocity information enhanced in higher frequency sway was obtained by the acoustic feedback which increased in volume when sway was larger. The system of Goodworth et al. [9] used a combination of pelvis angle and angular velocity [7,8]. They investigated which combinations of position and velocity feedback improved low frequency (<0.6Hz) sway [11] (No improvement for high frequency sway was noted with their system of vibration at the waist). Thus it remains to be investigated which combinations a vibrotactile can be used by patients and controls to improve high frequency sway. Assuming that in our study, patients used the acoustic information for correcting high frequency sway (>0.6Hz), no difficulties were apparent when patients in combined vibrotactile and auditory feedback into motor commands. The visual feedback we provided was only active for large sway angles and not available under eyes closed conditions.

This study may have implications for other patient groups when they receive biofeedback to reduce abnormal body sway. Here we have emphasized that vestibular loss subjects use the same in- and anti-phase movement strategies with feedback as healthy controls without feedback. We assume that the feedback substituted for the vestibular inputs that normally act to provide appropriate modulation of balance correcting strategies which are presumably triggered by proprioceptive inputs [25]. Another patient group, those with Parkinson’s disease, also showed improvement in roll sway with the identical type of feedback [31]. Whether the balance correcting strategies of these patients are similar to those of vestibular loss patients is not known. Lower-leg proprioceptive loss subjects use different balance correcting strategies [13]. For such patients, it is an open question whether these patients need to change their balance correcting strategies to those of healthy controls before they can be aided by the feedback schemes of this and other studies [4,7,8] or whether the feedback characteristics need to be changed appropriately to fit their abnormal balance correcting strategies.

## Conclusions

This study is the first to demonstrate how vestibular loss subjects achieve a reduction of sway during stance with prosthetic feedback of body sway. Unchanged movement strategies with reduced amplitudes are achieved with improved antagonistic muscle synergies at the lower legs and trunk. Thus both body movement and muscle measures could be explored as feedback variables for future prosthetic devices which aim to reduce sway of those with a tendency to fall.

## Abbreviations

BVL: Bilateral vestibular loss; HC: Healthy control; CNS: Central nervous system; CoM: Center of mass; LP: Low pass; BP: Band pass; HP: High pass; EO: Eyes open; EC: Eyes closed; ECN: Standing eyes closed on a normal floor; EOF: Standing eyes open on a foam floor; ECF: Standing eyes closed foam floor; PSD: Power spectral density.

## Competing interests

The authors report that J.H.J. Allum and F. Honegger worked as consultants for the company producing SwayStar™/BalanceFreedom™, part of the equipment used in this study.

## Authors’ contributions

FH developed the experimental instrumentation and software, designed the study, supervised experiments, analyzed and interpreted the data, and helped draft the manuscript. IMHA recruited and tested subjects and collected data, helped design the study, participated in analysis and wrote a preliminary draft. NGAE recruited and tested subjects and collected data, and participated in design. KST tested subjects and collected data, participated in design. JHJA designed the study and instrumentation, helped analyze and interpret the data and draft the manuscript. All authors read and approved the final manuscript.
